# Targeting MYC Dependence by Metabolic Inhibitors in Cancer

**DOI:** 10.3390/genes8040114

**Published:** 2017-03-31

**Authors:** Himalee S. Sabnis, Ranganatha R. Somasagara, Kevin D. Bunting

**Affiliations:** Department of Pediatrics, Division of Hematology/Oncology/BMT & Aflac Cancer and Blood Disorders Center, Emory University School of Medicine, 1760 Haygood Drive NE, HSRB E308, Atlanta, GA 30322, USA; hsabnis@emory.edu (H.S.S.); ranganatha.somasagara@emory.edu (R.R.S.)

**Keywords:** transcription factor, metabolism, cancer, signal transduction, combination therapy

## Abstract

*MYC* is a critical growth regulatory gene that is commonly overexpressed in a wide range of cancers. Therapeutic targeting of *MYC* transcriptional activity has long been a goal, but it has been difficult to achieve with drugs that directly block its DNA-binding ability. Additional approaches that exploit oncogene addiction are promising strategies against MYC-driven cancers. Also, drugs that target metabolic regulatory pathways and enzymes have potential for indirectly reducing MYC levels. Glucose metabolism and oxidative phosphorylation, which can be targeted by multiple agents, promote cell growth and *MYC* expression. Likewise, modulation of the signaling pathways and protein synthesis regulated by adenosine monophosphate-activated protein kinase (AMPK) and mechanistic target of rapamycin (mTOR) can also be an effective route for suppressing *MYC* translation. Furthermore, recent data suggest that metabolism of nucleotides, fatty acids and glutamine are exploited to alter MYC levels. Combination therapies offer potential new approaches to overcome metabolic plasticity caused by single agents. Although potential toxicities must be carefully controlled, new inhibitors currently being tested in clinical trials offer significant promise. Therefore, as both a downstream target of metabolism and an upstream regulator, MYC is a prominent central regulator of cancer metabolism. Exploiting metabolic vulnerabilities of MYC-driven cancers is an emerging research area with translational potential.

## 1. Introduction

The *c-MYC* proto-oncogene (referred to throughout as *MYC*) is a well-known driver of cancers. Cancer cells become reliant on the MYC-induced changes that occur in proliferation, metabolism, DNA repair, RNA splicing, and survival. Sustained expression of MYC is thus required to promote activity and interactions with binding partners needed for proper chromatin regulation and gene expression. Recent work using synthetic lethal screens has provided novel insight into the vulnerabilities of MYC-reliant cells. If these targets are perturbed, then MYC-expressing tumors are susceptible to oncogene-induced cell death [1,2]. Interestingly, targeting of non-oncogene addiction [3] is an established concept that is applicable for certain tumors such as those driven by MYC or RAS and presumed house-keeping functions upon which MYC is dependent, are being revealed as potential drug targets. Furthermore, changes in signaling and protein or nucleotide metabolism can reduce MYC protein levels, indicating a reciprocal relationship. In particular, metabolic stress required to support continued tumor growth is a potent vulnerability of tumors driven by MYC or RAS. The dynamic balance between oncogene-dependent and oncogene-independent tumor growth is explored further in this review, with a specific focus on the role of MYC in cancer cell biology. Since MYC activity is not as effectively influenced by drugs as kinases are, a variety of alternative options are discussed that could both target MYC dependencies as well as reduce MYC protein expression levels.

**Figure 1:**
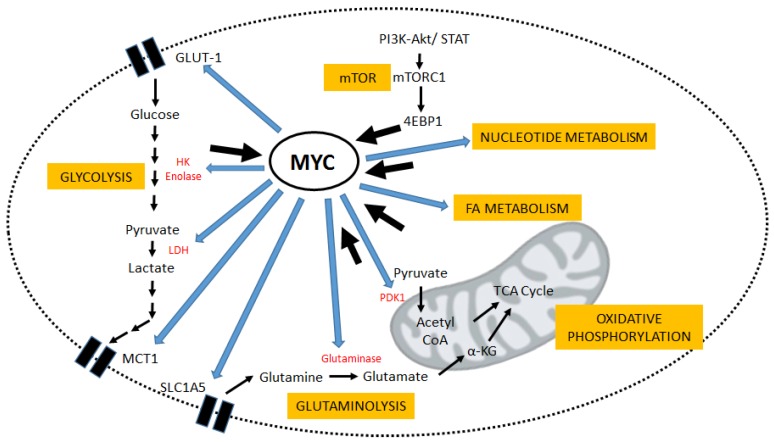
*MYC* is a centrally relevant gene that is both upstream and downstream of metabolic pathways. *MYC* is a regulator of glycolysis through targets genes that modulate both the uptake and breakdown of glucose to produce lactate. *MYC* also promotes glutamine metabolism as an alternative energy source. Control of nucleotide and fatty acid metabolism is also *MYC*-dependent. Positive roles for MYC protein expression are indicated by blue arrows. MYC is also downstream of many of these metabolic processes and targeting those pathways has therapeutic potential for suppression of MYC expression levels; this is significant for cancer therapy. The positive roles that these pathways play in MYC regulation are indicated by black arrows. Because of this central importance in cancer cell metabolism, MYC will continue to be a strong drug candidate which can be approached by multifaceted targeting. GLUT-1: Glucose Transporter-1; SLC1A5: Solute Carrier Family 1 member 5; MCT-1: Monocarboxylate transporter 1; LDH: Lactate Dehydrogenase; PI3K: Phosphatidyl-4,5-bisphosphate 3’ Kinase; AKT: Protein Kinase B; STAT: Signal Transducer and Activator of Transcription; 4EBP1: Eukaryotic Translation Initiation Factor 4E-binding Protein 1; PDK1: Pyruvate Dehydrogenase Kinase 1; α-KG: Alpha Ketoglutarate; TCA: Tricarboxylic Acid

Enhanced glycolysis within cancer cells, even in the presence of oxygen, was first described by Otto Warburg in the 1920s, and has since been known as the ‘Warburg Effect’ [4,5]. Advances in medical research in the last several decades have outlined tumor cell metabolism to be a far more complicated network of metabolic reprogramming, regulated at least partially by transcriptional control through oncogenes [6]. Glucose metabolism and oxidative phosphorylation, along with the metabolism of nucleotides, fatty acids, and glutamine, all seem to have important roles based on the conditions in the tumor micro-environment, with hypoxia-inducible genes and MYC target genes both being key players. MYC is both upstream and downstream of metabolism and thus is centrally relevant as a target in cancer therapy (Figure 1).

## 2. MYC and Glucose Metabolism

To comprehend the mechanism by which oncogenes control glucose metabolism, one must understand the promoter activity controlling the enzymes involved in glycolysis. Glucose levels in the blood influence hepatic metabolism of glucose via specific DNA sequences in the promoter region of target genes [7]. This sequence (5′-CACGTG-3′) called the carbohydrate response element (ChRE) binds transcription factors of the basic domain, helix-loop-helix, leucine zipper family [8]. Important rate-limiting glycolytic enzymes including hexokinase type II and l-type pyruvate kinase (PKlr), bear ChRE motifs in their promoter regions [9,10]. MYC is a basic-helix-loop-helix/leucine zipper protein that functions as a transcription factor after dimerizing with MYC-associated X (MAX); the dimer then binds to the DNA consensus core sequence CACGTG (or E box) [11]. The similarity between ChRE and the MYC binding sequences is interesting, since it suggests that glycolytic genes might be a direct target for the MYC dimer, and there is evidence to show that MYC is capable of influencing glycolysis via direct transcriptional activation of glycolytic enzymes as noted below. 

### 2.1. Transcriptional Control of Glycolytic Genes

Enolase (*ENO1*) and Glucose Transporter-1 (*GLUT-1*) were identified as direct MYC target genes in in vitro rat fibroblasts, and confirmed by further in vivo testing in murine livers [12]. Using MYC knockout and conditional cell lines, hexokinase II (*HK2*) was also identified as a direct MYC target gene [13]. Lactate dehydrogenase A (LDH-A), which is frequently overexpressed in numerous human cancers, was also identified as a MYC target gene in fibroblasts, and its over-expression was shown to be required for MYC-mediated transformation of human lymphoblastoid cells and Burkitt lymphoma cells [14]. Phylogenetic studies have confirmed the conservation of E boxes in *ENO1*, *HK2*, and *LDH-A* genes with high interspecies sequence identity, further validating them to be direct targets of MYC [15]. The relationship between MYC and LDH-A has been studied and characterized under various conditions. Normally under hypoxic conditions, expression of hypoxia-inducible-factor 1 (HIF-1) is increased. HIF-1 is a helix-loop-helix protein capable of binding to similar CACGTG or E box sequences like MYC, resulting in the transcriptional upregulation of enzymes involved in anaerobic glycolysis, including LDH-A [16]. Tumor cells typically exist in a micro-environment that is hypoxic, and express high levels of MYC [17,18]. In these conditions, both HIF-1 and MYC cooperate to further enhance their effects on glycolytic enzymes including LDH-A, resulting in glycolysis and the Warburg effect often seen in tumor cells [19]. In normal cells and under normoxic conditions, the effects on LDH-A are less pronounced, favoring the shift towards oxidative phosphorylation. The excess lactate produced in cancer cells can be toxic to the cell itself, and high levels result in over-expression of lactate transporters, specifically mono-carboxylate transporters (MCTs) [20]. This results in acidification of the tumor microenvironment, which may contribute to tumor invasion and metastasis [21]. Recently, MCT1 was shown to be a MYC target and inhibition of MCT1 resulted in intracellular lactate accumulation in tumor cells, and eventual cell death [22]. In addition, MYC transcriptionally represses microRNAs miR29a and miR29c, which results in enhanced expression of MCT1 on tumor cells [23]. 

### 2.2. Indirect Transcriptional Control of Glycolytic Genes

There is some evidence to suggest that MYC acts indirectly through other transcription factors to influence the degree of glycolysis within cancer cells [24]. A specific transcription factor identified in the early 2000s was the carbohydrate response element binding protein (ChREBP) [25]. This protein functions as a heterodimer and encodes a basic helix-loop-helix leucine zipper transcription factor that is capable of binding to ChRE motifs in the promoter regions of glycolytic genes, including pyruvate kinase in hepatocytes. Its activity is enhanced after consumption of a high carbohydrate diet and it is modulated by glucose levels rather than lactate production. The presence of MYC has been shown to be necessary for ChREBP-dependent transcription of l-type pyruvate kinase in relation to serum glucose levels; however, the exact binding site for MYC has not been identified [26]. In H1LC rat hepatoma cells, antisense *MYC* mRNA and a dominant negative MAX protein decreased both l-type pyruvate kinase and glucose-6-phosphatase levels [27]. In the same study, adenoviral overexpression of MYC induced glucose-6-phosphatase even in the absence of glucose. A complex comprising of hepatocyte nuclear factor 4α (HNF-4α) and 1α (HNF-1α) along with ChREBP and cAMP response binding protein (CBP) is necessary for the transcription of Pklr to proceed and MYC may work by recruiting all members to the promoter site and/or by preparing the chromatin to facilitate the interaction of all the complex members [28].

## 3. Targeting MYC Dependence through Glucose Metabolism

See Table 1 for a list of glucose metabolism inhibitors described in this section.

### 3.1. GLUT-1 Inhibitors

MYC has been shown to transcriptionally upregulate the expression of GLUT-1 in rat fibroblasts [12]. Though there is no direct evidence for whether GLUT-1 inhibitors affect MYC levels, multiple GLUT-1 inhibitors are currently being studied. These include fasentin, STF-31 and WZB117, which have shown reduction in tumor growth and size in breast, renal, and lung cancer models [29,30,31].

### 3.2. Hexokinase Inhibitors

*a*. 3-Bromopyruvate
  3-Bromopyruvate (3BP) was initially described as a metabolic inhibitor in the early 1960s, but has since been studied extensively for its effect on glycolysis and glycolytic enzymes. In a liver cancer model, 3BP was shown to effectively inhibit hexokinase 2, and inhibit glycolysis, facilitating death of hepatoma cells [32]. 3BP is a structural analog of pyruvic acid and is thought to be taken up by cells via MCTs [33]. High levels of MYC expression in tumor cells drives the overexpression of MCTs, enabling efficient uptake of 3BP and enhanced lethality of the tumor cells in response to this drug [23]. In addition to HK2, 3BP effectively inhibits the glyceraldehyde 3-phosphate dehydrogenase (GAPDH) enzyme, another important enzyme in glycolysis, resulting in significant depletion of cellular ATP and cell death [34,35].
*b*. 2-Deoxyglucose
  In the early 1950s, 2-deoxy-d-glucose (2DG) was shown to inhibit both aerobic and anaerobic glycolysis in rat tumor tissue [36]. It is a synthetic glucose analog, in which the C-2-hydroxyl group is replaced by hydrogen, and for the last several decades it has been used extensively to study tumor cell metabolism. While the effects of 2DG on glycolysis are the focus of most studies, this agent acts in several different ways to kill cancer cells [37]. 2DG enters cells via GLUTs and is phosphorylated by HK to form 2-Deoxy-d-glucose-6-phosphate (2DG-6-P) which then inhibits both HK and phosphoglucose isomerase (PGI) activity, thereby decreasing glycolysis and reducing the ATP/AMP ratio in cells [38,39]. Increased levels of HIF-1 and MYC can induce resistance to 2DG via upregulating the levels of glycolytic enzymes [40]. Drugs like methylprednisolone, cisplatin or ABT-737 which reduce HIF-1 and MYC levels can synergize with 2DG to inhibit cell proliferation and induce apoptosis [41,42,43].

### 3.3. Metformin

Metformin, a biguanide used widely in treatment for type 2 diabetes, has garnered tremendous interest over the past fifty years in its role as an anti-cancer agent [44]. The direct target for metformin is not clearly defined, however it is believed to act via inhibition of Complex I of the mitochondrial respiratory chain [45]. This results in significant ATP depletion and lowering of the ATP/AMP ratio, with subsequent AMPK activation. AMPK activation further stimulates upregulation of glucose transporters, and favors a switch toward glycolysis and increased lactate production. mechanistic target of rapamycin (mTOR) is inhibited via AMPK-mediated activation of the Tuberous Sclerosis Complex (TSC)1/2 complex [46]. In prostate cancer mouse models expressing high levels of MYC, treatment with metformin has been shown to decrease MYC levels both in vivo and in vitro, inhibiting the growth of prostate cancer cells while minimally inhibiting growth of normal prostatic epithelial cells [47]. Metformin has also been shown to exert its effects via microRNAs involved in cellular metabolism. Upregulation of AMPK and mir33a results in down-regulation of MYC in breast cancer models [48]. It is important to note that cellular concentrations of glutamine and glucose play an important role in the effects seen with metformin [49]. Both glutamine and glucose levels intracellularly are modulated by MYC, thus making the metabolic effects of metformin closely related to MYC expression. 

### 3.4. LDH Inhibitors

For tumor cells that rely on glycolysis to maintain their ATP production, use of LDH inhibitors could potentially disrupt this dependence, making tumors sensitive to apoptosis and cell death. LDH activity is also important for maintaining NAD/NADH levels intracellularly [50]. Silent information regulator 1 (SIRT1/Sirtuin 1) is a NAD(+)-dependent deacetylase that can affect MYC function. MYC activates SIRT1, which in turn promotes MYC function [51].

*a*  Galloflavin
  Galloflavin, identified as a LDH inhibitor in 2012, was capable of inhibiting both LDH A and B isoforms of the enzyme [52]. It is capable of blocking glycolysis and inducing cell death. LDH inhibition also results in lower NAD levels and lower activity of SIRT1, thereby decreasing MYC protein levels. In Burkitt lymphoma cells, the down regulation of MYC results in inhibition of lymphoma cell growth [53].
*b*  Other LDH inhibitors
  Gossypol, Oxamate and FX11 are some of the LDH inhibitors currently being studied for their efficacy in various cancer models [54].

### 3.5. Pyruvate Dehydrogenase Kinase Inhibitor—Dichloroacetate 

Dichloroacetate is a Pyruvate Dehydrogenase Kinase 1 (PDK1) inhibitor that is capable of inhibiting glycolysis and inducing apoptosis in cells dependent on glycolysis as the primary ATP source [55]. This property of PDK1 inhibition has been used in non-malignant conditions such as mitochondrial disorders causing lactic acidosis as well as pulmonary hypertension [56]. It is also being studied as a single agent and with other drug combinations in numerous cancer models [57]. 

### 3.6. Other Glucose Metabolism Inhibitor Targeting MYC

Diclofenac, a non-steroidal anti-inflammatory drug, has recently shown the ability to inhibit GLUT-1, LDH-A, and MCT1 expression in cancer cell lines, along with decreased MYC activity, resulting in reduced cell proliferation and tumor growth [58].

## 4. MYC and mTOR Pathway

mTOR is a central regulator of mammalian metabolic and physiological processes [59]. Recent studies highlight the properties of the highly conserved mTOR Ser/Thr kinases in the regulation of diverse signals, including nutrients, growth factors, energy and stress control, cell growth, proliferation, survival and metabolism [60]. The mTOR proteins play important roles as critical regulators of energy homeostasis in cells. The inhibition of mTOR downregulates the production of ATP in cells, and ATP depletion is a characteristic of structurally diverse MYC inhibitors [61]. Several drugs are on the market that downregulate metabolic activity by inhibiting mTOR. In diverse cancer models, mTOR represents a central target for cancer therapy [62].

### 4.1. Post Translational Regulation of MYC by mTOR

*MYC* is an oncogene that is expressed in a number of cancer models, but therapies that target *MYC* directly are not clinically available. The oncogenic activity of *MYC* directly depends on its capacity to increase protein synthesis. Hence, inhibiting enhanced protein synthesis is a plausible strategy for treating *MYC*-driven human cancers. Unfortunately, MYC itself is not easily affected with drugs, due to lack of enzymatic activity and a drug-interacting pocket, which would otherwise be a good target for small molecule inhibitors [2]. However, since inhibition of mTOR has effects on nutrient uptake by tumor cells and alters their metabolism, targeting mTOR signaling to regulate MYC protein is a potential approach [63]. mTOR regulates protein synthesis through phosphorylation of the tumor suppressor eukaryotic translation initiation factor 4E (eIF4E) binding protein 1 (4EBP1), and p70S6 kinase (p70S6K1/2). 4EBP1 inhibits translation initiation by binding to the mRNA cap recognition element of the translation initiation complex protein eIF-4E. mTOR phosphorylation of 4EBP1 leads to the dissociation of eIF-4E, thus increasing translation initiation complex interactions with the mRNA 5′ cap [64]. Hence, blocking the hyper-activation of eIF4E by dephosphorylation of 4EBP1 through mTOR inhibition is critically required for the inhibition of protein synthesis and hindering tumorigenesis [65]. mTOR stabilizes the MYC protein, as well as inducing *MYC* translation [66]. Recent studies show that the oncogenic effects of MYC are due to increased protein synthesis, leading to cell proliferation. Protein synthesis is not only enhanced by the transcriptional activity of MYC but also by activating the mTOR-dependent phosphorylation of 4EBP1. Since the MYC protein is short-lived, with a half-life of 15 to 30 min, it requires continuous synthesis to maintain its level, and thus the inhibition of cap-dependent translation would rapidly decrease the MYC protein level [67]. Hence, targeting translational effects on MYC is one approach to suppress MYC protein activity. In another study, Cianfanelli et al. showed that MYC protein regulation is controlled by mTOR through the AMBRA/PP2 complex protein [68].

### 4.2. Targeting MYC Dependence through mTOR Inhibition

mTOR inhibitors target MYC protein in several different ways that are summarized in Table 2.

Rapamycin is a traditional mTOR inhibitor. Rapamycin is a macrocyclic antibiotic produced in the bacterium *Streptomyces hygroscopicus*. Initially, rapamycin was developed as a potent antifungal agent, but was later found to be a potent mTOR inhibitor. Rapamycin inhibits the mTOR complex 1 (mTORC1) by destabilizing the mTOR-Raptor complex. Rapamycin binds to a highly conserved cytoplasmic receptor FK506-binding protein-12 (FKBP12) [83]. This complex formation inhibits the kinase activity of the TOR protein subfamily. Rapamycin treatment affects different cells which are derived from multiple tumor models, like rhabdomyosarcoma, neuroblastoma, glioblastoma, small-cell lung carcinoma, osteosarcoma, pancreatic carcinoma, renal cell carcinoma, Ewing sarcoma, prostate cancer, and breast cancer [69]. Potent inhibitor effects of rapamycin are observed with IL-2-induced T cell proliferation (Inhibitory Concentration (IC)_50_ = 0.05 nM) [84]. Currently, three different analogs of rapamycin include CCI-779 (temsirolimus), RAD001 (everolimus) and AP23573 (deforolimus) which are available for use in humans [85].

Since the traditional mTOR inhibitor, rapamycin, has poor solubility that compromises its potential as an intravenous agent in humans, newer agents have been developed. The Cell Cycle Inhibitor-779 (CCI-779) is an ester analog of rapamycin that was developed as a novel mTOR inhibitor with increased solubility [72]. CCI-779 demonstrated antitumor activity alone or in combination with other cytotoxic agents in a variety of human cancer models such as gliomas, rhabdomyosarcoma, medulloblastoma, head, neck, prostate, pancreatic, and breast cancer cells [86]. CCI-779 inhibits the cell growth in breast cancer cell culture with IC_50_ values in the nanomolar range. CCI-779 also inhibits MYC protein expression in breast cancer cells with a decrease in the phosphorylated protein level of 4EBP1 [64]. Intraperitoneal injections of CCI-779 also induce significant dose-dependent responses against subcutaneous growth of myeloma cells [72].

RAD001 (everolimus) is a functionally similar derivative of rapamycin, and is an allosteric inhibitor of mTOR [75]. Rapamycin as an oral drug with poor bioavailability, led to the development of this new analog RAD001 with improved bioavailability [85]. AZD8055 is a novel ATP-competitive mTORC1/mTORC2 kinase inhibitor. Rapamycin and analogs (rapalogs) have limited clinical utility, due to negative feedback and lack of inhibition of mTORC2, leading to the activation of AKT and subsequent attenuation of rapalog effects on mTORC1. AZD8055 potentially inhibits the phosphorylation of mTORC1 substrates p70S6K and 4EBP1, along with the mTORC2 substrate AKT and its downstream proteins. Importantly, AZD8055 significantly decreases the phosphorylation of 4EBP1 on the rapamycin insensitive Thr37/46 sites, with potential inhibition of cap-dependent translation at low nanomolar concentrations. AZD8055 is a potential growth inhibitor in broad range of tumor types. AZD8055 is currently being tested in phase I clinical trials [87,88]. Notably, it has been found to strongly reduce the MYC protein level through mTOR downregulation in A459 lung cancer cells [78].

Icariside II is a natural flavonoid compound, which inhibits phosphorylation of mTOR activity in sarcoma cells. Icariside II inhibits aberrant energy homeostasis in sarcoma cells, as evidenced by reduction of energy production by glycolysis and energy consumption by mRNA translation. Icariside II inhibits MYC protein expression through downregulation of the mTOR-4EBP1 axis [60].

MTI-31 is a novel mTOR kinase inhibitor. This inhibitor targets mTOR signaling in several relevant tumor models at an oral dose of 5 mg/kg in tumor-bearing mice. ATP citrate lyase (ACL) is a critical enzyme in glucose-derived de novo lipogenesis that converts citrate to acetyl-CoA. Unlike rapamycin, MTI-31 specifically inhibits the Ser-455 phosphorylation of ACL, which is important in cellular de novo lipid synthesis. The dysregulation of mTOR-ACL is a tumorigenic mechanism important for fueling cancer cell growth and survival. MTI-31 in combination with etomoxir, a fatty acid oxidation inhibitor, suppresses the MYC protein level and cell growth in breast cancer cells [89].

MLN0128, a PP242 derivative, belongs to a new class of second generation mTOR inhibitors, which includes additional compounds by Wyeth-Ayerst and AstraZeneca. This small molecule agent induces cell death in renal cell carcinoma by suppressing mTOR-mediated 4EBP1 phosphorylation. MLN0128 also showed potential inhibition of proliferation of Merkel cell carcinoma cells and robust increases in antitumor activity when treated along with JQ1, a BRD4 (bromodomain 4) inhibitor [79]. MLN0128 decreases the level proteins like 4EBP1, p-S6K1, and MYC through translational regulation. MLN0128, is currently being tested in phase I clinical trials.

OSI-027 is an mTOR inhibitor which inhibits mTOR activity by decreasing the phosphorylation of 4EBP1. OSI-027 treatment inhibits proliferation and induces apoptosis in wide variety of lymphoid cells including Jurkat, Nalm-6, Molt-4 and SeAX cells [80]. OSI-027 treatment in Jurkat cells decreases MYC translation levels by increasing the dephosphorylation of 4EBP1 and downregulating 4EBP1-dependent translational activity [80].

The combination treatment of PP242, a rapamycin kinase inhibitor and IRES-J007, MYC-IRES translation inhibitor significantly reduces tumor growth in glioblastoma (GBM) xenografts in mice. The first generation allosteric mTOR inhibitors such as rapamycin and mTOR analogs were unsuccessful as a single treatment in GBM treatment, because of loss in feedback regulation and activation of AKT [89]. The newer generations of kinase inhibitors have greater potential in GBM treatment as they downregulate the negative feedback activation of AKT. This combination treatment significantly reduces MYC mRNA translation [82].

### 4.3. Dual PI3K and mTOR Inhibitors

BEZ235 is an orally administered dual PI3K and mTOR kinase inhibitor. The combination of BEZ235, along with vincristine treatment, enhanced apoptosis in Lck-Dlx5 lymphoma cells by downregulation of both AKT activity and MYC expression [90]. BEZ235 downregulates MYC translation through increasing the amount of 4EBP1 associated with eIF4G by mTOR inhibition [91]. The combination of BEZ235 and DNA damage response inhibitors also induced more apoptosis through p53-independent mechanisms in MYC-driven lymphomas [92]. RAD001 and BEZ235 combination treatments induced a strong anti-proliferative effect as compared to the individual drugs in non-small cell lung cancer [75].

PI-103 is another dual PI3K and mTOR kinase inhibitor. The inhibition of mTOR by rapalogs increases AKT activity and promotes cell growth by phosphorylation of the upstream negative regulator of mTOR, the TSC1/TSC2 complex [93]. The combination of both PI-103 and RAD001 blocked the RAD001 induced stimulation of AKT. The inhibitory effect of p-AKT with this drug combination is greater than the effect from the single drugs. The combination treatment also showed greater activity over single agents in inhibiting the phosphorylation of 4EBP1 and downregulation of the MYC protein level in different cancer models [94].

SPS-7 is a triazole-based small molecule, which inhibits mTOR through PI3K/AKT signaling inhibition. In prostate cancer, SPS-7 induced an inhibitory effect on mTOR, which downregulated mTOR-dependent phosphorylation of 4EBP1. The inhibition of PI3K/AKT/mTOR activity led to decreased MYC protein levels through destabilization [67].

### 4.4. Other Agents with mTOR Inhibitory Activity

MS-275 is a dual inhibitor that blocks both mTOR activity, as well as histone deacetylation in both AML and promyelocytic leukemia cells. The combination treatment of MS-275 with the rapamycin analog RAD001 (enhanced MS-275 mediated growth inhibition and apoptosis through downregulation of MYC protein. This combination treatment also induced terminal differentiation in both HL-60 and NB-4 acute promyelocytic leukemia cells. Treatment with both RAD001 (5 mg/kg b.wt) and MS-275 (10 mg/kg b.wt) inhibited proliferation of HL-60 tumor xenografts in nude mice without detectable adverse effects [73].

RTP801 is a negative regulator of the mTOR protein. ATRA induces RTP801, which inhibits mTOR signaling, with decreases in the levels of p-p70S6K and p-4EBP1, leading to the suppression of MYC protein expression in AML cells. The combination treatment of ATRA and RAD001 significantly induced growth arrest and differentiation of AML cells. The combination of both RAD001 and ATRA induced the down regulation of MYC [66].

## 5. MYC and Nucleotide and Fatty Acid Metabolism

MYC is a regulator of diverse metabolic processes that include many types of macromolecules. MYC expression is regulated downstream of both nucleotide and lipid metabolism. Nucleotide metabolism has classically been studied in cancer biology, and nucleotide-targeted therapies have been a mainstay in conventional chemotherapy, due to its selective toxicity to rapidly dividing tumor cells. One of the most common attributes of successful MYC inhibition in cancer cells is the depletion of intracellular ATP [61]. The combination of triplex-forming oligonucleotides targeting MYC was also found to synergize with anti-metabolite chemotherapy agents, such as gemcitabine [95]. Tight linkages between MYC expression and deoxynucleotide pools establish a critical connection, upon which the success of many chemotherapy agents lies. In melanoma, MYC directly regulates genes in melanocytes, and targeting these nucleotide pools has an effect similar to reduction in MYC protein expression [96].

Lipid metabolism is an emerging area of cancer biology. Studies using a myristoylated AKT or an inducible *MYC* transgene, have demonstrated an important role for MYC in promoting aerobic glycolysis. Importantly, depletion of fatty acids or glutamine was sufficient to sensitize to glycolysis inhibition resulting in cell death [97]. Inhibition of MYC results in mitochondrial dysfunction, and a resulting accumulation of lipid droplets in tumor cells [98]. This type of accumulation has also been described for other proteins such as the carnitine palmoyltransferase 1C (CPT1c) [99]. The gene encoding for one of the two sphingosine kinase isoenzymes, sphingosine kinase 2 (SPHK2), catalyzes the phosphorylation of sphingosine into sphingosine-1-phosphate. SPHK2 promotes acute lymphoblastic leukemia through a MYC-dependent mechanism. Inhibition of SPHK2 results in reduced MYC expression, and has demonstrated pre-clinical efficacy [100].

### 5.1. Targeting MYC Dependence through Nucleotide Metabolism

6-Benzylthioinosine (6-BT) is a promising new cancer drug that can deplete ATP and induce myeloid differentiation [101]. 6-BT was identified from a small molecule screen for compounds from the NCI repository that can induce myeloid differentiation, as measured by nitroblue tetrazolium color change in a high-throughput assay [101]. Out of this screen, 6-BT was identified as a molecule similar to all-trans-retinoic acid (ATRA) in the ability to differentiate HL-60 cells. However, 6-BT possessed additional leukemia cell specificity, the ability to inhibit ent1 mediated nucleoside transport, and the ability to kill a subset of AML samples tested. In addition, 6-BT was able to suppress expression of BCL2 over a 3–5-day time course, suggesting that part of its apoptosis induction mechanism may involve suppression of survival signals. However, the molecular mechanism for the cytotoxicity induction was incompletely understood. 

There are several interesting features of this drug that were not characterized in the initial study, and which point toward potential efficacy in phospho-signal transducer and activator of transcription 5 (pSTAT5)^+^ myeloid leukemias. Cell lines with the lowest IC_50_ were MV-411 (2 µM) and HNT34 (0.5 µM), both of which are characterized by activating tyrosine kinase mutations responsible for driving STAT5 phosphorylation. MV-411 cells are FLT3-ITD^+^ AML and HNT34 cells are BCR-ABL^+^ Chronic Myelomonocytic Leukemia (CMML) cells that have evolved to AML. Therefore, 6-BT may be especially useful for inducing cytotoxicity in the phospho-STAT5^+^ subset of AML. In contrast, the cell lines examined that had higher IC_50_ were HL-60 (30 µM) and OCI-AML3 (>100 µM), which are not known to carry tyrosine kinase-activating mutations. Instead of cytotoxicity, the HL-60 and OCIA-ML3 cells had ATP depletion, growth arrest, and terminal differentiation. Therefore, ATP depletion is tightly correlated with differentiation induction, but due to the induction of cell death in MV-411 cells, it was not possible to assess the ATP depletion at 24 h.

Amino acids stimulate Rag guanosine triphosphatases (GTPases) to bind to GTP and interact with mTORC1. Rag proteins comprise a family of four related small GTPases that are required for mTORC1 activity. 6-BT must be phosphorylated to be active, and the phosphorylated 6-BT is structurally identical to the 6-mercaptopurine anti-metabolite 6-methylthioinosine monophosphate (MeTIMP), differing only in the addition of a benzene ring. 6-BT might effectively suppress PPAR-amido-transferase, which is required for de novo purine biosynthesis, leading to reduction in guanine and adenine nucleotides. 6-BT could effectively suppress mTORC1 and downstream 4EBP1, primarily through an AMPK-independent mechanism. Suppression of mTORC1 mediated signaling has been reported in mycophenolic acid (MPA) treated cells. MPA promotes differentiation of HL-60 cells [102,103,104], but in IL-3 dependent cell lines 32D, FL5.12, and BaF3 cells, it can induce apoptosis through guanine nucleotide depletion [105], and interestingly it can synergize with Imatinib in cells engineered to express p185 BCR-ABL from a retroviral vector [106]. MPA was not only able to inhibit mTORC1, it also induced caspase 3-dependent apoptosis, reduced p70S6K and 4EBP1 phosphorylation, and mediated decreases in MYC and cyclin D protein translation. These effects were due to depletion of GTP since add-back of guanosine but not adenine, was able to reverse the effects.

It is worthy to note that although 6-BT and its analogs have been shown to have cytotoxic activity against the parasitic protozoan *Toxoplasma gondii*, this is due to their recognition as a subversive substrate for adenosine kinase in this parasite, something not seen in mammalian cells [107,108,109]. Therefore, a role in adenosine salvage is not a likely explanation for its anti-cancer activity. However, another potential advantage of 6-BT is leukemia cell specificity. 6-BT has an IC_50_ of >100 µM in mouse embryonic fibroblasts, normal human bone marrow, human umbilical vein endothelial cells (HUVEC), and human mononuclear cells. Although 6-BT can bind to the equilibrative nucleoside transporter (ENT1) and inhibit its function, the 6-BT uptake mechanism may be due to an alternative transporter, since ent1 inhibition does not block its phosphorylation and activity. Adenosine transport through the concentrative nucleotide transporter (CNT2) is one possible mechanism of uptake. However, it is important to note the apparent leukemia cell specificity of 6-BT, which may improve the therapeutic index for this agent.

### 5.2. Targeting MYC Dependence through Fatty Acid Metabolism

Targeting fatty acid oxidation is also a potential approach for MYC inhibition. It has been recently reported that in a MYC-driven triple-negative breast cancer model, inhibition of fatty acid oxidation function through a MYC-mediated mechanism suppressed the growth of MYC-overexpressing cells [110]. This linkage highlights a critical therapeutic approach. In pancreatic cancer, targeting SPHK2 was especially potent at targeting E2F and MYC, and this therapy worked particularly well in combination with gemcitabine [111]. Interestingly, the SPHK2 inhibitor reduced MYC protein expression in a dose-dependent manner. SPHK2 inhibition has also been tested in multiple myeloma, alone or in combination with the BH3 mimetic ABT-737 [112]. Synergistic responses were observed with downregulation of MYC and MCL1 expression levels, and significant suppression of tumor cell growth in xenograft mouse models. In colorectal cancer, SPHK2 was upregulated, and knockdown by small interfering RNA inhibited invasion and proliferation. Overall, there is growing evidence of cross-talk between fatty acid oxidation and MYC.

## 6. MYC and Glutamine Metabolism

While the role of glucose and the Warburg effect is important in tumor metabolism, it does not provide the only source of ATP that tumor cells need. Glutamine, typically a non-essential amino acid, has been shown to be an important source of energy for cancer cells [113]. During periods of rapid proliferation, glutamine demand falls behind supply and it becomes an essential amino acid [114]. Glutamine can be utilized by cells in different ways, and its conversion to glutamate provides a prime source for energy production through the Krebs cycle. Glutamine is converted to glutamate by glutaminase, an enzyme that is highly expressed in tumor cells [115]. Glutamate is then converted to α-ketoglutarate (α-KG) via transaminases or glutamate dehydrogenase, and undergoes further oxidation via Krebs cycle generating ATP. Early evidence for MYC control of glutamine metabolism was first described by Yuneva et al in 2007 [116]. A year later, Wise et al. demonstrated that glutamine functions as a vital source of energy production in tumor cells expressing high levels of MYC, leading to a condition they defined as glutamine addiction [117]. In their model, glutamine maintained mitochondrial function and viability, and MYC increased the surface expression of glutamine transporters. MYC also transcriptionally represses microRNA miR-23a and miR-23b, which increases the expression of glutaminase, resulting in greater conversion of glutamine to glutamate [118]. Mitochondrial protein p32 is a regulator of tumor metabolism, and plays an important role in maintaining oxidative phosphorylation [119]. p32 has been shown to be a direct target of MYC, and is important for MYC control of glutamine metabolism [120]. Recently, MYC has also been shown to directly stimulate glutamine synthesis via transcriptionally upregulating thymine DNA glycosylase (TDG), which in turn demethylates the promoter for glutamate synthetase (GS), allowing for increased expression of the enzyme. GS synthesizes the formation of glutamine from glutamate and ammonia [121].

Thus, via control of both glycolysis and glutaminolysis, MYC functions as an essential regulator of cancer cell metabolism, allowing for tumor cells to meet their metabolic needs depending on the availability of glucose and/or glutamine as a substrate for glycolysis or oxidative phosphorylation. In addition to MYC’s ability to control glutamine metabolism directly, other regulatory pathways exist to monitor MYC activity. mTORC1 via protein S6 Kinase 1 functions to regulate the effect of MYC on glutaminase [70]. Mitochondrial protein SIRT4 represses the effects of MYC on glutamine metabolism, and can synergize with glycolytic inhibitors and induce cell death [122]. Lactate levels themselves can serve to modulate the degree of glycolysis versus glutaminolysis within cells via HIF-1α and MYC activation [123]. Within liver tumors, inhibitor of differentiation 1 (ID1), and within colorectal cancer cells, *N*-Myc downstream regulated gene 2 (NDRG2), function as suppressors of MYC function that further impact cellular metabolism [124,125]. In prostate cancer, prostate cancer gene expression marker 1 (PCGEM1) is a long coding RNA that can interact with MYC directly and enhance its activity [126]. Among the 14-3-3 proteins, 14-3-3σ is capable of enhancing ubiquitination and degradation of MYC, and functions as a key regulator of cell metabolism in breast cancer cells [127]. Currently, multiple strategies to target glutamine metabolism are being tested in pre-clinical studies and in early clinical trials, which indirectly serve to inhibit the effects of MYC on glutamine metabolism [128].

## 7. Combination Therapies Targeting MYC

### 7.1. Arsenic Trioxide and Dichloroacetate

Arsenic trioxide inhibits cytochrome c oxidase, a component of complex IV of the mitochondrial respiratory chain [129]. When combined with DCA which inhibits glycolysis, the combination effectively decreased levels of MYC and HIF-1α as well as pro-survival protein Bcl-2, resulting in cell death [130].

### 7.2. 6-BT and Metformin

Metformin is capable of effectively inhibiting mitochondrial respiration and decreasing ATP levels intracellularly. A decrease in ATP production favors AMPK activation, enhances glycolysis, and causes mTOR inhibition [45]. Glycolysis and increased ATP production provide an important mechanism for cells to escape the effects of metformin. Recently, we have shown that 6-BT is capable of significantly decreasing the glycolytic flux, especially in AML cells with FLT3-ITD mutations [131]. This was also accompanied by decrease in GLUT-1 mRNA levels. The specific target for 6-BT however, still remains to be determined, but it may act via blockading glucose transport, similar to its analog, nitrobenzylthioinosine (NBTI) [132]. A combination of 6-BT and metformin resulted in marked synergistic cell death within FLT3-ITD positive cell lines. This was accompanied by a decrease in MYC mRNA and protein levels.

## 8. Summary and Future Directions

It is well established that among genes that control normal and cancer cell metabolism, MYC is undoubtedly a pivotal player. Oncogenic pathways involved in malignant transformation often result in MYC activation, which can then induce metabolic and structural changes within cells, promoting proliferation, increased survival and resistance to apoptosis. The tightly controlled expression of MYC, its short half-life, and critical role in normal cell metabolism makes direct targeting of MYC a challenge for anti-cancer therapeutics. In this review we covered various approaches to targeting MYC dependencies that are already at various stages of preclinical and clinical development. However, additional approaches may be developed in the future as a way to overcome current limitations. The challenges and opportunities for new approaches have been discussed here.

Direct targeting of MYC has typically involved either a siRNA-based approach using Dicer-substrate small interfering RNA (DCR)-MYC, or the use of bromodomain and extra-terminal (BET) inhibitors that target proteins with bromodomains. RNA interference (RNAi) has the unique ability to knock down specific genes within cancer cells, but typically delivery of RNAi to cancer cells has been a challenge [133]. Development of newer vehicles of siRNA delivery, such as lipid nanoparticles, has been instrumental in overcoming this challenge. Currently, two Phase 1/2 clinical trials are underway to test DCR-MYC in patients with solid tumors and hematopoietic malignancies (NCT02110563, NCT02314052). BET proteins function as transcriptional regulators of MYC, and their inhibitors disrupt the interaction between BET proteins and MYC, which may result in reduced cell proliferation in cancer [134]. Currently they are being tested in hematologic and lymphoid malignancies, including multiple myeloma and other advanced cancers (NCT01943851, NCT02711137, NCT02158858, NCT01949883, NCT02431260, NCT02157636). Future clinical studies will require improved delivery methods and more sophisticated nanoparticle delivery, possibly including receptor-targeted nanoparticles. 

Additional small molecules may be developed based on protein-protein interaction (PPi) screens. A recent study demonstrated that PPi hubs can be identified for a variety of cancer-related genes, including *MYC*, *STK11*, *RASSF1*, and *CDK4* [135]. By identifying this type of OncoPPi network, it may be possible to inform new therapy development for MYC dependence. Focus on downstream and metabolic inhibitors also remains an attractive option for indirectly affecting MYC expression and decreasing its transcriptional activity. Metabolomic profiling of various cancers has shown that each cancer type can possess unique metabolic features that distinguish them from other cancer types, which can then be further exploited to develop specific targeted therapies. Inhibitors discussed in this review focus on targeting glucose, protein, nucleotide, and glutamine metabolism, each of which may play varying roles, depending on the cancer cell type. MYC dictates acetyl-CoA abundance and fate [136]. In the absence of glucose and glutamine, acetyl-CoA is generated from acetate that is liberated from histone proteins, which in addition to their role in epigenetic regulation, can also serve as a sink for storing acetate. The acetyl-CoA can be used for energy in tumor cells, and has been demonstrated in a human glioblastoma model [137,138]. Targeting MYC might be able to target this newly identified bioenergetic pathway and/or disrupt utilization of this backup storage mechanism.

Significant tumor heterogeneity might exist between patients, and therefore a more personalized assessment of MYC dependence may be needed. Patient profiling through use of synthetic lethality screens might be required in order to select the most optimal patients for metabolism inhibitors. Additionally, tools such as nanoproteomic assays [139] might be developed using the NanoPro 1000 to monitor signaling and MYC target gene expression in rare tumor cell populations. Nanoproteomic assays have already been developed and tested for several cancers, and in the context of metabolism, assays for AKT1/2/3 and 4EBP1 are able to quantify signaling in acute myeloid leukemia cells [139,140].

Irrespective of the mechanisms by which MYC dependence is targeted, the end goal involves decreasing cellular ATP production and inducing cell death. It is possible that changes seen in MYC expression following indirect metabolic inhibition may be secondary to apoptosis, and this is currently being studied. Lastly, there is a potential role for eventually combining both direct and indirect inhibitors of MYC to develop more novel anti-cancer therapies. In summary, innovative molecular and cellular approaches are required to target MYC dependence in cancer, and because of its high value as a therapeutic target, these will continue to attract research efforts in the future.

## Figures and Tables

**Table 1:** Inhibitors targeting glucose metabolism.

Target in Glucose Metabolism	Inhibitor Name	References	Clinical Testing
GLUT-1	Fasentin	[30,31,32]	Preclinical phase only, No current clinical trials
	STF-31		
	WZB117		
Hexokinase	3-Bromopyruvate	[33,34]	3-BP: Preclinical only, 2-DG: Multiple phase 1/2 clinical trials in lung, prostate, breast tumors
	2-Deoxyglucose	[37,38,39,40,41]	
GAPDH	3-Bromopyruvate	[35,36]	Preclinical phase only
Phosphoglucose Isomerase	2-Deoxyglucose	[39,40]	Multiple phase 1/2 clinical trials in lung, prostate, breast tumors
AMPK	Metformin	[46,47]	Multiple phase 1 through 3 clinical trials in lung, pancreatic, ovarian tumors, leukemias
LDH	Galloflavin	[53]	Galloflavin: Preclinical only, Gossypol: Multiple phase 1/2 clinical trials in lung, prostate, brain, leukemias and lymphomas Oxamate & FX11: Preclinical only
	Gossypol	[55]	
	Oxamate	[55]	
	FX11	[55]	
PDK1	Dichloroacetate	[56]	Phase 1 clinical trials in breast, lung, brain, head & neck tumors
Unknown Target	Diclofenac	[59]	No specific cancer therapy trials

GLUT1: Glucose Transporter 1; GAPDH: Glyceraldehyde 3-Phosphate Dehydrogenase; AMPK: 5’-Adenosine Monophosphate-Activated Protein Kinase; LDH: Lactate Dehydrogenase; PDK1: Pyruvate Dehydrogenase Kinase 1; BP: Bromopyruvate; DG: Deoxyglucose

**Table 2:** Mechanistic Target of Rapamycin (mTOR) inhibitors and their mode of inhibition.

mTOR Inhibitor	Mechanism of Action	Protein Inhibition *	References	Current Clinical Timeline
Rapamycin	Destabilizes the mTOR-Raptor complex	MYC	[69,70]	Phase 1 through 4 clinical trials in multiple cancers (solid organ, hematopoietic cancers)
CCI-779 (Temsirolimus)		Cyclin-D1, Cyclin-D3, MYC	[64,71,72]	
RAD001 (Everolimus)		MYC, Cyclin D1	[66,73,74]	
Icariside II	mTOR Kinase inhibitor	MYC	[60]	Preclinical testing only
BEZ235	mTOR Kinase inhibitor	Cyclin A, Cyclin D1, Parp, Caspase 3, MYC	[75,76]	Phase 1 through 3 clinical trials in breast, prostate, renal tumors
MTI-31	mTOR Kinase inhibitor	p-Akt, Cyclin D1, MYC	[77]	Preclinical testing only
AZD8055	mTOR Kinase inhibitor	MYC, Mcl-1, c-Jun, Cyclin E	[78]	Phase 1/2 clinical trials in advanced solid tumors, lymphomas etc.
MLN0128 (INK128)	mTOR Kinase inhibitor	4EBP1, p-S6K1, MYC	[79,80]	Phase 1/2 clinical trials in thyroid, lung, endometrial, breast, myeloma, lymphoma etc.
PI-103	mTOR Kinase inhibitor	MYC, Cyclin D3, PI3K, p-Akt	[77,81]	Preclinical testing only
PP242	mTOR Kinase inhibitor	MYC, Cyclin D1	[82]	Preclinical testing only
OSI-027	mTOR Kinase inhibitor	MYC	[80]	Phase 1 clinical trial in advanced solid tumors & lymphoma

* Indirect translational down regulation of different proteins by mTOR inhibitors as per the previous literature. MCL1: Myeloid Leukemia Cell Differentiation Protein 1; 4EBP1: Eukaryotic Translation Initiation Factor 4E-Binding Protein 1; S6K: Ribosomal Protein S6 Kinase Beta-1; PI3K: Phosphatidyl-4,5-bisphosphate 3’ Kinase; AKT: Protein Kinase B.
